# InAs/Si Hetero-Junction Nanotube Tunnel Transistors

**DOI:** 10.1038/srep09843

**Published:** 2015-04-29

**Authors:** Amir N. Hanna, Hossain M. Fahad, Muhammad M. Hussain

**Affiliations:** 1 Integrated Nanotechnology Lab, Electrical Engineering, Computer Electrical and Mathematical Sciences & Engineering Division, King Abdullah University of Science and Technology, Thuwal 23955-6900, Saudi Arabia

## Abstract

Hetero-structure tunnel junctions in non-planar gate-all-around nanowire (GAA NW) tunnel FETs (TFETs) have shown significant enhancement in ‘ON’ state tunnel current over their all-silicon counterpart. Here we show the unique concept of nanotube TFET in a hetero-structure configuration that is capable of much higher drive current as opposed to that of GAA NW TFETs.Through the use of inner/outer core-shell gates, a single III-V hetero-structured nanotube TFET leverages physically larger tunneling area while achieving higher driver current (I_ON_) and saving real estates by eliminating arraying requirement. Numerical simulations has shown that a 10 nm thin nanotube TFET with a 100 nm core gate has a 5×normalized output current compared to a 10 nm diameter GAA NW TFET.

Tunneling field effect transistors (TFETs) offer interesting opportunities to address two major challenges faced by aggressively scaled conventional CMOS technology: (i) the increasing difficulty in scaling the supply voltage (V_DD_), and (ii) minimizing the leakage currents that degrade the I_ON_/I_OFF_ switching ratio. Both of them lead to more power consumption in devices whereas we need exactly the opposite (energy efficiency) for wide-range deployment of ultra-mobile computation capability. As the transistor gate length is reduced, improved performance, requires the supply voltage, V_DD_, and simultaneously the threshold voltage, V_T_, to be lowered to maintain a high overdrive factor (V_DD_ – V_T_)^1^. Doing this, however, exponentially increases the ‘OFF’ state leakage current (I_OFF_) due to a physical limitation commonly referred to as the 60 mV/dec sub-threshold slope (SS) bottleneck. This is inherent in all current generation electronics that utilizes CMOS transistors with over-the-barrier charge transport physics.

Additionally, transistor off-state power dissipation is considered to be empirically proportional to^1^:


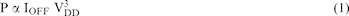
In order to reduce power consumption, reducing V_DD_ is absolutely critical, which in turn demands devices with steep SS enabling faster turn on at low supply voltages. Contrary to classical MOSFETs, where charge carriers are thermally injected by lowering an energy barrier, the primary transport mechanism in a TFET is inter-band tunneling, where charge carriers transfer from one energy band into another at a heavily doped p^+^/n^+^ junction. In a TFET, inter-band tunneling can be switched “ON” and “OFF” abruptly by controlling the band bending in the channel region using gate-to-source bias, V_gs_. This can be realized in a reverse-biased p-i-n structure, where asymmetric doping is used to suppress ambipolar transport^1^. While all-silicon TFETs have been studied rigorously using technology drive current boosters such as the use of a high-κ gate dielectric, abrupt doping profiles at the tunnel junction, ultra-thin body, higher source doping, a double gate, a gate oxide aligned with the intrinsic region, and a shorter intrinsic region (and gate) length, I_ON_ of just 100 µA/µm have been achieved^2^. One of the more efficient ways to radically improve I_ON_ is by using a low band gap material as the source injector. Here, the smaller effective mass of charge carriers increases the tunneling probability, according to the triangular Wentzel–Kramer–Brillouin (WKB) approximation^3^,^4^. Potential source material candidates for N/PMOS TFETs are Germanium (Ge) and Indium Arsenide (InAs), respectively. Simulation studies using the above materials in a hetero-structure have shown I_ON_ values of 244 µA/µm and 83 µA/µm for Ge and InAs NTFET and PTFET, respectively, which corresponded to I_ON_ enhancements by factors > 400× and > 100×, respectively, over their all-Si planar counterparts^1^. Combining this with new and unique non-planar architectures opens up new opportunities for TFETs that are on-par with traditional Boltzmann transistors. Table 1 summarizes some recent state-of-the-art III-V source TFET demonstrations.

We have recently shown the unique advantages of vertical nanotube architecture with core-shell gates for improved drive current capability compared to nanowires on silicon. Inspired by carbon nanotubes, the nanotube architecture mimics the gate all-around nanowire (GAA NW) devices by having an outer (shell) gate, as well as, an inner (core) gate inside the nanowire making it a hollow cylindrical structure. When compared to arrays of nanowires, the nanotube architecture outperforms in terms of drive current capability, CV/I metric (i.e. intrinsic gate delay), power consumption, and area efficiency^5^,^6^,^7^,^8^. In this paper we present a hetero-structure Si/InAs p-channel TFET device concept that combines the advantages of a low band-gap source injector and inherent high drive current advantage in NTFET (Figure 1).

Tunneling in TFET devices is governed by the inter-band tunneling probability across the tunneling barrier, which is typically calculated using WKB approximation^9^:


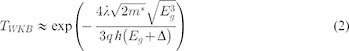
Where m* is the effective mass, E_g_ is the band gap, λ is the screening tunneling length, and Δ*Φ* is the potential difference between the source valence band and channel conduction bands. From this simple triangular approximation, we can see that that the band gap (E_g_), the effective carrier mass (m*) and the screening tunneling length (λ) should be minimized to increase the tunneling probability. While E_g_ and m* are material dependent parameters, λ depends on other parameters such as the device geometry, doping profiles and gate capacitance. A small λ value would result in a strong modulation of the channel bands by the gate^1^. It has been shown that the highest tunneling rate and hence lowest λ values were found for the gate-all-around (GAA) architecture for a 10 nm diameter nanowire, while ultra-thin body (UTB) double gate FETs has shown comparatively higher λ values^10^. Planar UTBs have the highest λ values^10^. Because λ is also sensitive to gate capacitance, tunneling probability can also be enhanced by using high-κ gate dielectrics, as well as, small channel body thickness. Also, the abruptness of the doping profile at the tunnel junction is also important to control *Δ*Φ. In order to minimize the tunneling barrier, the high source doping level must fall off to the intrinsic channel in as short a width as possible. Typically this requires a change in the doping concentration of about 4–5 orders of magnitude within a distance of only a few nanometers^1^.

With the above in mind, we hypothesize that the nanotube TFET’s excellent electrostatic control would enable steep turn on characteristics, while maintaining low I_OFF_ values comparable to NW TFET. This transistor architecture in conjunction with a low band gap source material in a hetero-structure configuration would enable a higher inter-band tunneling rate, when compared to all-silicon TFET structure. For this reason, InAs was chosen as a source material, for a p-type TFET application, since theoretically it has a band gap of 0.385 eV and electron effective mass of 0.026 m_o_^11^. Also, a recent experimental demonstration of InAs NW on Si(111) has shown the possibility of growing NW without misfit dislocation for diameters less than 20 nm, which is attributed to the reduction of strain field owing to the nanometer-scale footprint of NW^12^. The authors found that the hetero-structure with small diameter NW possesses fewer misfit dislocations, which quantitatively suppress trap assisted tunneling via dislocation levels, and has pure band-to-band tunneling as the dominant tunneling process. The highest InAs normalized current by the same group is 0.5 mA/µm for n-type using p+ Si source, and Zn doped n+ InAs channel^12^.

## Results

(Figure 2) shows the energy band diagram of the simulated p-channel nanotube TFET. (Figure 3(a)) compares the normalized I_ds_-V_gs_ characteristics of a 10 nm thin NTFET (with 100 nm inner core-gate diameter, CG_dia_) and 10 nm diameter NWFET at V_dd_ = 1V. Transfer characteristics at lower supply voltages are shown in Supporting Figure S1^13^. We have used the NW circumference (πd), where d is the NW diameter, in the case of the NT we have used average circumference 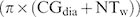, where CG_dia_ and NT_w_ are the nanotube core-gate diameter and thickness respectively. As it can be seen the nanotube architecture has 5× higher normalized current output than that of the GAA NW architecture. Both architectures provide I_ON_/I_OFF_ of more than 10^5^. We have compared threshold voltage, V_T_, values for both devices, and they were −0.58V and −0.63V for the NT and NW TFETs, respectively, showing a difference of 0.05V, using the constant current method defining V_T_ at a normalized current of I_ds_ = 10^−7^A/μm^14^. However, we have also used the proposed threshold definition by Boucart *et al.* arguing that TFET threshold voltages can be physically defined based on the saturation of the barrier width narrowing with respect to V_gs_^15^. According to the new definition of threshold votlage, V_TG_, is the voltage at which the first derivative of the transconductance, *g_m_*, shows a maxima with respect to V_gs_^15^. We have found that V_TG_ = −0.73 and −0.72V for the NT and NW TFETs, respectively, which shows that the two devices have similar threshold voltage values. Both threshold voltage extraction methods are shown in Supporting Figure S2. So, this confirms the fairness of the comparison as both devices show similar normalized I_OFF_, and V_TG_ values.

Figure 3(b) compares the SS values of the 10 nm NT and NW FETs. Both architectures show SS values less than 60 mV/dec over 5 decades^1^. However, the NW architecture TFET shows lower point SS values as low as 25 mV/dec, while the lowest SS values for the NTFET is ~ 40 mV/dec. We have shown before in the past this is due to the ultimate electrostatic control in the GAA architecture^6^. However, smaller drain bias has been experimentally reported to yield lower point and average SS^16^, as is also shown in Supporting Figure S3 at V_ds_ = −0.8V, where the NT TFET shows a minimum point SS of 22 mV/dec, and the NW TFET shows a minimum if 23 mV/dec. The reason for this is that lower V_ds _provides in principle lower I_ON min_, the I_ON_ value at the knee of the SS swing, thus also the average SS slope would be reduced. We have also compared the non-normalized “ON” current of the 10, 20, 30 nm NW TFETs with the 10 nm NT TFET in (Figure 4(a)). The 10 nm NT TFET shows a non-normalized I_ON_ ~ 0.32 mA, while the NW TFETs show I_ON_ ~ 5.9×10^−6^A, 2.51×10^−5^A, 5.31×10^−5^A for the 10, 20 and 30 nm NWs, respectively. This means that the 10 nm NTFET shows 54×, 13× and 6× increase in drive current over that of the 10, 20 and 30 nm diameter NW TFETs. As for SS, (Figure 4(b)) shows that only 10 and 20 nm NW TFET and the 10 nm NT TFET can achieve sub 60 mV/dec SS. So, having a small diameter NW is essential to maintain a low SS.

## Discussion

In order to supply high drive current while maintaining small SS, arraying small diameter NWs is inevitable. However, this would come at the expense of chip area and off-state leakage as will be seen the following sections. Additionally, the low “OFF” current characteristic of the TFET would be lost as arraying would cause, at the least, multiplying the “OFF” state leakage current by the number of NWs in the array needed to supply the same “ON” current as one NT. Therefore, in the case of 54 NW array of 10 nm diameter NWs, the leakage would be at least 13× higher compared to a single 10 nm NT. Finally, although it could be argued that arraying could boost the “ON” current value, sensitivity of parameters like threshold voltage, for example, variations in NW width could lead to degradation of the SS swing for a large array of devices^17^. Recent demonstrations of sub 60 mV/dec of have been for all silicon single NW p and n-type TFET of diameter < 20 nm which supplies a maximum ON current in the nA regime and a normalized “ON” current of 1.2 µA/µm^18^,^19^. However, when an array of TFETs is tested sub 60 mV/dec SS have been demonstrated for currents as low as 0.01 µA/µm^20^. Also, degraded SS slope was noticed for higher drain current giving a maximum I_ON _ = 64 μA/μm at V_DD _ = 1.0 V and at a higher gate bias V_GS_ = 2 V. On the other hand, a SS of 52 mV/dec have been shown at an even higher I_ON _ = 100 μA/μm at V_DD _ = 1.0 V and V_gs _ = 1 V for all-silicon single gated SOI based TFETs with vertical self-aligned top gate structure supplying and for 70 nm thick SOI with 2 nm Effective Oxide Thickness (EOT)^21^. Even higher drain currents have been shown for double gate strained-Ge hetero-structure TFET with a drive current of 300 μA/μm at a SS of 50 mV/dec^22^. That is why we think the NT architecture could be an excellent candidate for as a vertical structure that resembles double gate structure and could provide a higher integration density compared to the NW structure.

To consider the scalability of the nanotube architecture, we studied the non-normalized drain current as a function of the inner core-gate diameter (Figure 5(a)). An important observation here is that the core-gate contact scaling tunes the on-state drive performance without compromising the sub-threshold swing, as can be seen from (Figure 5(b)). This becomes a competitive technology option compared to GAA NWFETs. To fully comprehend this concept, consider the top-down plan-view chip layout perspective of a single nanotube and an array of GAA nanowires. (Figure 6(a)). In vertical GAA nanowire technology, maintaining a small nanowire pitch (NW_pitch_) ensures high integration density and drivability. In order to compete with this, the core-gate diameter (CG_dia_) of the nanotube should be highly scalable just like the nanowire pitch. One pragmatic approach to investigate this is by studying the device dimension scalability effects on chip area. Using the 2013 International Technology Roadmap for Semiconductors (ITRS) Overall Roadmap Technology Characteristics (ORTC), scalable parameters for current generation FinFET technology are adapted here, which are summarized in Table 2. In this comparison, the nanowire pitch is assumed to be equal to the fin half-pitch and the nanotube core-gate diameter is assumed to be equal to contact/via size as specified in the 2013 ORTC target for future technology nodes. The contact/via size scaling is considered equal to the M1 metal half-pitch. The other parameters in the comparison are the nanowire diameter (NW_dia_) and the nanotube thickness (NT_w_), both of which are assumed equal to the fin width target given in Table 2. From (Figure 4(a)), a 100 nm core-gate diameter, 10 nm thin InAs/Si nanotube has greater than 10× higher non-normalized drive capability compared to a single 10 nm diameter GAA InAs/Si nanowire. To achieve similar performance levels as the nanotube, more than 10× nanowires need to be stacked in an array. Using the above parameters and assuming that the normalized drive current scales linearly with channel thickness (NT_w_ and NW_dia_) in both the nanotube and nanowire architecture, chip-area estimates of the single nanotube and a 10× nanowire array are carried out at different technology nodes using simple analytical calculations. As it can be seen from (Figure 6(b)), the contact/via scaling at the moment is comparable to the nanowire-pitch. But at around the 7 nm technology node (2017), the pitch scaling will start to become more aggressive. However, even after this and considering the fact that large nanowire arrays are required to achieve similar drivability as a single nanotube, the InAs/Si nanotube architecture will outperform InAs/Si GAA nanowire arrays at the extreme scaling limit in terms of chip area consumed.

We also compared the BTBT generation rate of both the 10 nm nanotube and 10 and 20 nm GAA NW TFETs in (Figure 7(a)). Color maps of BTBT generation rate and SRH Recombination rate of the 10 nm NT, 10 nm NW and 20 nm NW TFETs are provided in Supporting Figures S4, S5 and S6, showing the cross sections in the middle of the channel along which we made the measurements. The 10 nm diameter nanowire shows a slightly higher peak BTBT generation of 1.6×10^32^ (cm^−3^s^−1^), when compared to the 10 nm thick nanotube, showing ~1.5×10^32^ (cm^−3^s^−1^), due to the shorter tunneling length, λ, for the nanowire architecture. Similar peak BTBT generation rate is expected as it has been theoretically shown that the differences in the scaling tunneling length, λ, between the GAA and double gate architectures, which resembles the NT Core-Shell gates architecture,reduces for body thickness ≤ 10 nm and almost diminishes for body thickness of 5 nm^10^. On the other hand, the larger diameter nanowire (20 nm) shows lower peak tunneling rate of 5.53×10^31^ (cm^−3^s^−1^) due to larger body thickness leading to higher tunneling length, λ, values. However, one major difference between the two architectures is the distance over which BTBT generation is significant away from the Si/InAs interface. For the case of the NT, we observe that BTBT generation rate is higher over a larger distance, almost 7 nm into the Silicon channel, when compared to both the 10 nm and 20 nm NW, as shown in (Figure 7(a)). This is an indication of higher lateral tunneling across the Si-InAs interface for the NT architecture when compared to the NW architecture, which could explain the higher normalized current. This reason for this is that when analyzing the band diagrams for the 10 nm NT and NW TFETs, shown in Figure S7, it was found that the gradient of the holes quasi Fermi level is higher for the NT TFET as we move from the Si/InAs interface into the Si channel. This is important since the BTBT tunneling is proportional to the valence band gradient, according to the mode used. So, a higher gradient corresponds to lower scanning tunneling length, λ. In addition, lateral tunneling is fundamentally limited by the inversion layer thickness of in the ‘ON’ state, and thus is sensitive to the Channel/Source interface cross sectional area.

On the other hand, the 10 nm NW TFET is shows higher BTBT generation rate when moving away from the interface into the InAs source, indicating higher vertical tunneling within the source, when compared to both the 10 nm NT and 20 nm NW TFETs. Although higher vertical tunneling is desired in hetero-structure TFETs for increasing the drive current^23^, it could also lead to higher Shockley-Reed-Hall (SRH) recombination in the small direct band gap InAs source. When analyzing the SRH recombination rate in (Figure 7(b)), it was found that the peak SRH recombination rate for the 10 nm NW is almost an order of magnitude higher than the 10 nm NT TFET, as well as, the 20 nm NW TFET. To get a quantitative sense of the effect of BTBT generation and SRH recombination rates on the drive current, we analyzed the integrated area under the curve for (Figure 7(a–b)). For the 10 nm NT BTBT generation curve, the area under the curve was found to be 8.4% higher than that of the 10 nm NW curve, and 45% higher than that of the 20 nm NW curve. This can partially account for the larger non-normalized current seen for 10 NT TFET, 54×, compared to that of the 10 nm nanowire TFET. The nanotube has larger available cross sectional area for tunneling when compared to the 10 nm nanowire. Since a 100 nm core-gate diameter nanotube with 10 nm thickness has 44× the cross sectional area of a 10 nm nanowire. Hence, doing a back-of-the-envelope multiplication the extra 8.4% in volumetric band-band generation by the additional cross sectional area gives 47.7× the anticipated increase in the current. The area under the SRH recombination curve for the 10 nm NW 8.7× of that of the 10 nm NT and 12× of that of the 20 nm NW.This demonstrates the potential of the nanotube transistor architecture to radically enhance the drive current capability of tunnel FETs to values comparable to state-of-the-art CMOS due to both higher BTBT generation rate and lower SRH recombination rate when compared to NW architecture at the same body thickness.

We precluded discussion over the fabrication of nanotube TFET as it is out of the scope of this paper and can be found in our pertinent work. With the advent of III-V channel material growth on silicon^12^,^24^, the nanotube device formation using bottom-up approach is very much possible.

## Conclusion

We have presented the advantages of nanotube architecture with inner/outer core-shell gates for hetero-structure (Si/InAs) TFET application, when compared to gate-all-around nanowire based TFETs. 3D device simulations have shown that a p-channel nanotube TFET is able to outperform nanowire arrays, while preserving chip area and at comparable SS values. We believe the nanotube architecture combined with hetero-structure III-V/IV material systems holds a great promise for high performance, ultra-low power consumer computing applications.

## Methods

**Figure 1:**
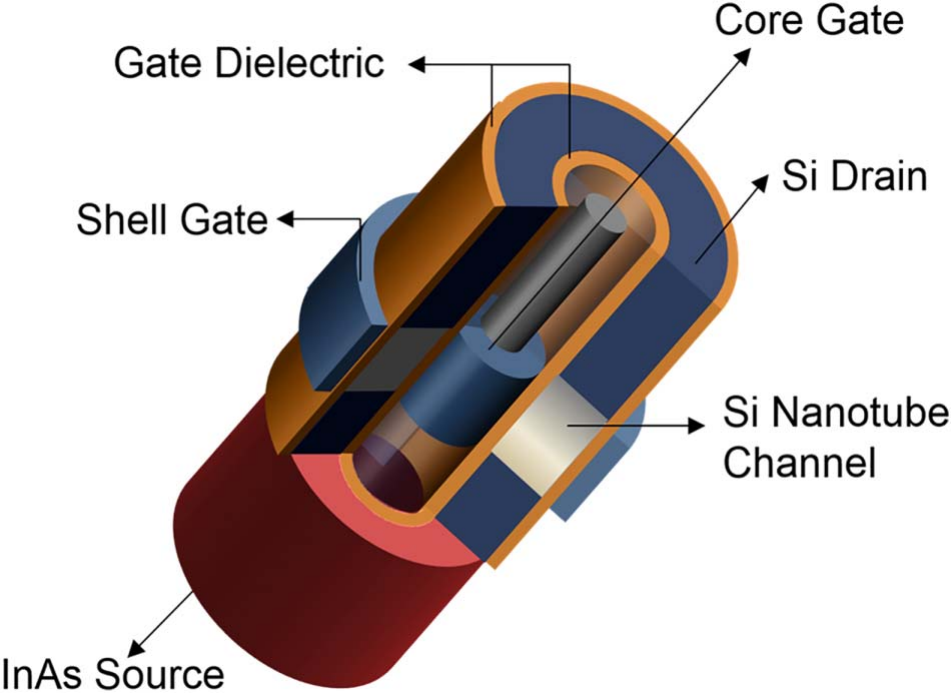
Schematic of the Nanotube (NT) architecture.

**Figure 2:**
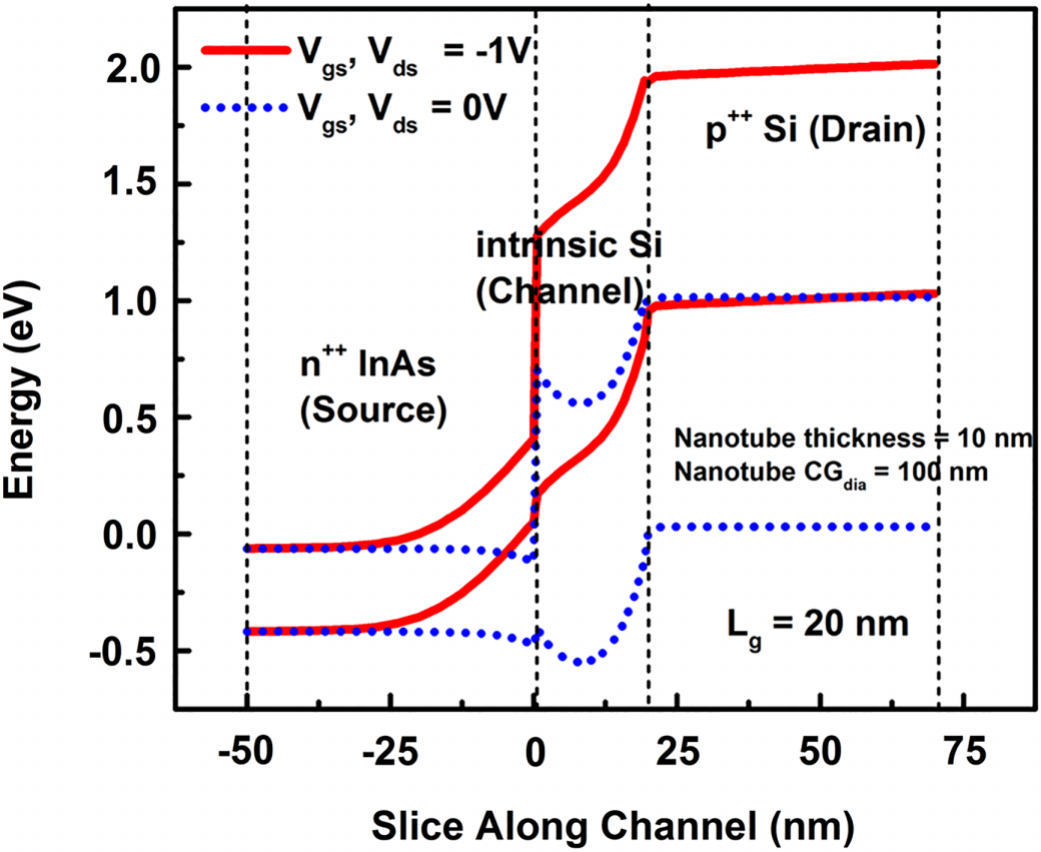
Band diagram of the P-type nanotube architecture TFET showing both ON state OFF states.

**Figure 3:**
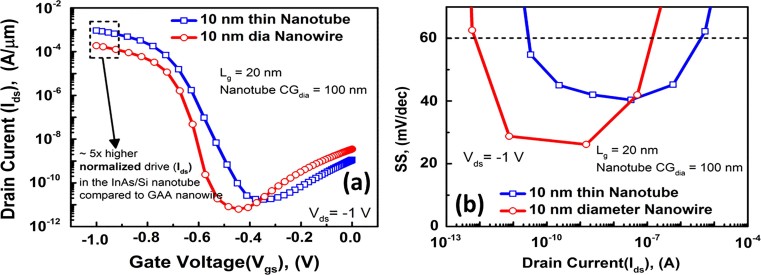
(a) Normalized I_ds_-V_gs_ characteristics of a 10 nm diameter NW and 10 nm thin NT p-channel TFETs; and (b) Sub-threshold Slope (SS) for the NT and NW TFET showing sub 60 mV/dec for more than 5 orders of magnitude of current.

**Figure 4:**
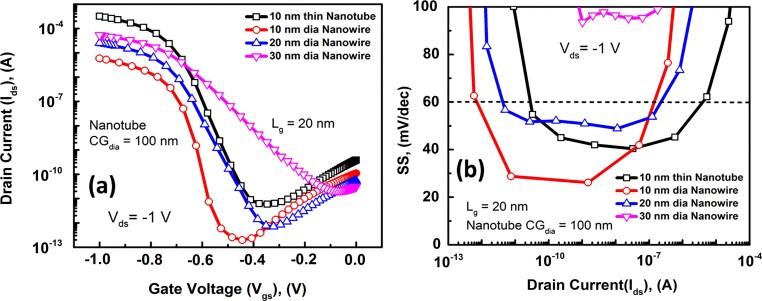
(a) Transfer (I_ds_-V_gs_) characteristics of NWs of 10, 20, 30, 40 and 50 nm diameter and 10 nm NT; and (b) SS comparison between 10 nm thick NT and 10, 20, and 30 nm diameter NW TFETs.

**Figure 5:**
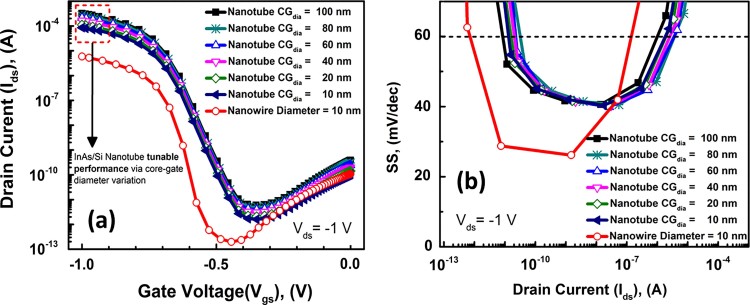
Non-normalized NT drive current (a) and SS (b) as a function of inner core gate diameter.

**Figure 6:**
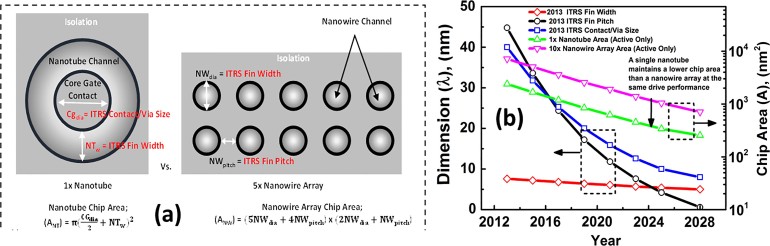
(a) Illustrated top-down plan-view comparison of between; and (b) chip area comparison using ITRS predicted parameters between a single nanotube device and an array of 10 × GAA nanowires.

**Figure 7:**
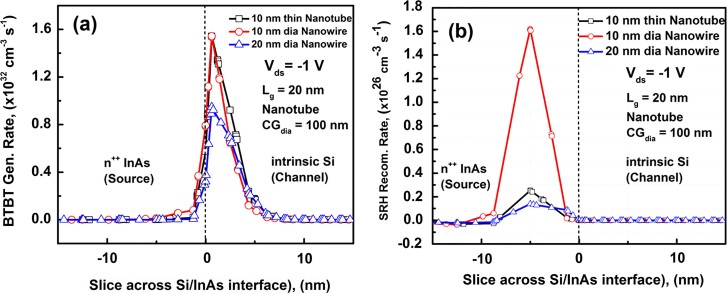
(a) BTBT Generation Rate; and (b) SRH Recombination Rate for the 10 nm NT and 10, 20 nm NWs as function of the distance from the Si/InAs interface.

To study the benefits of a nanotube architecture over a nanowire on a hetero-structure Si/InAs TFET platform, 3D simulations of a NT (Figure 1) and GAA NW TFET using Synopsys™ using the dynamic nonlocal path BTBT model^11^,^24^,^25^. For this case, indirect BTBT model was assumed as Si is an indirect bandgap material. The tunneling path for this model is calculated as a straight line straight line with its direction opposite to the gradient of the valence band and ends conduction band. Both devices are compared for a gate length (L_g_) of 20 nm. Silicon drain is p-doped with acceptor active concentration N_A_ = 1×10^20^ cm^−3^, while an intrinsic channel is used. The InAs source was used with n-doping with donor active concentration N_D_ = 1 × 10^18^ cm^−3^, both typical to the previously demonstrated device^24^. Both the nanotube thickness and nanowire diameter are kept at 10 nm. The gate metal in both devices has a work function of 4.53 eV, and a nitride gate dielectric is assumed with an (effective oxide thickness) EOT of 0.5 nm. A dynamic nonlocal band-to-band (BTB) tunneling model is utilized in conjunction with Shockley–Reed–Hall recombination and drift–diffusion physics. The band-to-band tunneling (BTBT) parameters, ‘A’ and ‘B’ for silicon are 4 × 10^14^ cm^−3^ s^−1^ and 1.9 × 10^7^ V cm^−1^ respectively^24^,^25^. While for InAs the BTBT parameters were taken as 9 × 10^19^ cm^−3^ s^−1^ and 1.3×10^6^ V cm^−1^ respectively^11^.

This comparative simulation study does not include any gate overlap with the source, assumes an ideal interface with no defects due to strain and takes into account trap-assisted tunneling due to dopants induced defect levels. However, it does not take into account bandoffsets due to strain, quantum confinement effects, and multiple valley BTBT effects. The dynamic nonlocal BTBT model uses the Kane two-band model which is a simple two-band model capable of including one conduction band and one valence band and it is formulated as two coupled Schrodinger-like equations for the conduction-band and valence-band envelope functions. The coupling term is treated by the k•p perturbation method, which gives the solutions of the single electron Schrodinger equation in the neighborhood of the bottom of the conduction band and the top of the valence bands, where most of the electrons and holes, respectively, are concentrated. The valence band and conduction band dispersion relationship are assumed to be bulk-like. This simulation framework has been previously utilized in the literature for simulating tunneling in hetero-structures for both Si/InAs Esaki diodes and TFETs^11^,^25^. The nanotube TFET has a silicon channel thickness of 10 nm and an inner core gate diameter (CG_dia_) of 100 nm. All contacts are assumed to be Ohmic with zero contact resistance.

## Author Contributions

MMH conceived the idea and directed the experiment. ANH carried out the experiment. HMF provided experimental support. All analyzed the data and wrote the paper.

## Supplementary Material

Supplementary Information Supporting info

## Figures and Tables

**Table 1:** Summary of state-of-the-art III-V TFET demonstrations

Affiliation	Ref.	Year	Technology	Material System	I_on_(A) per nanowire	I_on_(µA /µm)	SS (mV/dec)
Penn State	[26]	2010	Non planar single gate	In_0.53_Ga_0.47_As	NA	4×10^−1^	100–216
Intel	[27]	2011	Planar	InGaAs	NA	~ 7	60
UC Berkeley	[11]	2011	Planar	InAs	NA	0.5	190
Univ. of Notre Dame	[28]	2011	Vertical InGaAs/ InP	InGaAs	NA	20	130
IBM	[29]	2011	GAA NW	InAs/Si	10^−7^	0.4	220
UT Austin	[30]	2011	Vertical single gate	In_0.7_Ga_0.3_As	NA	40	84–380
IBM	[24]	2012	GAA NW	InAs/Si	Not reported	2.4	150
Hokkaido Univ	[31]	2012	Vertical hetero NW	InAs/Si	Not reported	~ 0.005	21 / 114
Univ. of Notre Dame	[32]	2012	Planar single gate	GaSb-InAs	NA	180	200–400
Univ. of Notre Dame	[33]	2012	Planar single gate	InP-InGaAs	NA	20	93–310
Penn State	[34]	2012	Vertical single gate	GaAsSb-InGaAs	NA	135	230–350
Univ. of Notre Dame	[35]	2012	Planar single gate	AlGaSb-InAs	NA	78	125–470
Hokkaido Univ	[12]	2013	Vertical hetero NW	InAs/Si	Not reported	1	21
MIT	[36]	2013	Vertical single gate	InGa_0.53_As_0.47_-GaAs_0.5_Sb_0.5_	NA	0.5	140

**Table 2:** 2013 ITRS ORTC FinFET SCALING PARAMETERS

Technology Node	Year	MPU/ASIC M1 ½-Pitch (nm)	Fin ½-Pitch (nm)	Fin Width (nm)
16/14	2013	40	30	7.6
10	2015	31.8	24	7.2
7	2017	25.3	19	6.8
5	2019	20	15	6.4
3.5	2021	15.9	12	6.1
2.5	2023	12.6	9.5	5.7
1.8	2025	10	7.5	5.4
