# Supplementary material for: InAs/Si Hetero-Junction Nanotube Tunnel Transistors
Source: Sci Rep. 2015 Apr 29;5:9843. doi: 10.1038/srep09843 (PMC4413881; doi:10.1038/srep09843)
Supplement: Supplementary Information — Supporting info [file srep09843-s1.pdf]

InAs/Si Hetero-Junction Nanotube Tunnel Transistors

Amir N. Hanna[1], Hossain M. Fahad[1], and Muhammad M. Hussain[1,*]

[1]Integrated Nanotechnology Lab, Electrical Engineering, Computer Electrical and Mathematical Sciences & Engineering Division, King Abdullah University of Science and Technology, Thuwal 23955-6900, Saudi Arabia.

Supplementary Figures

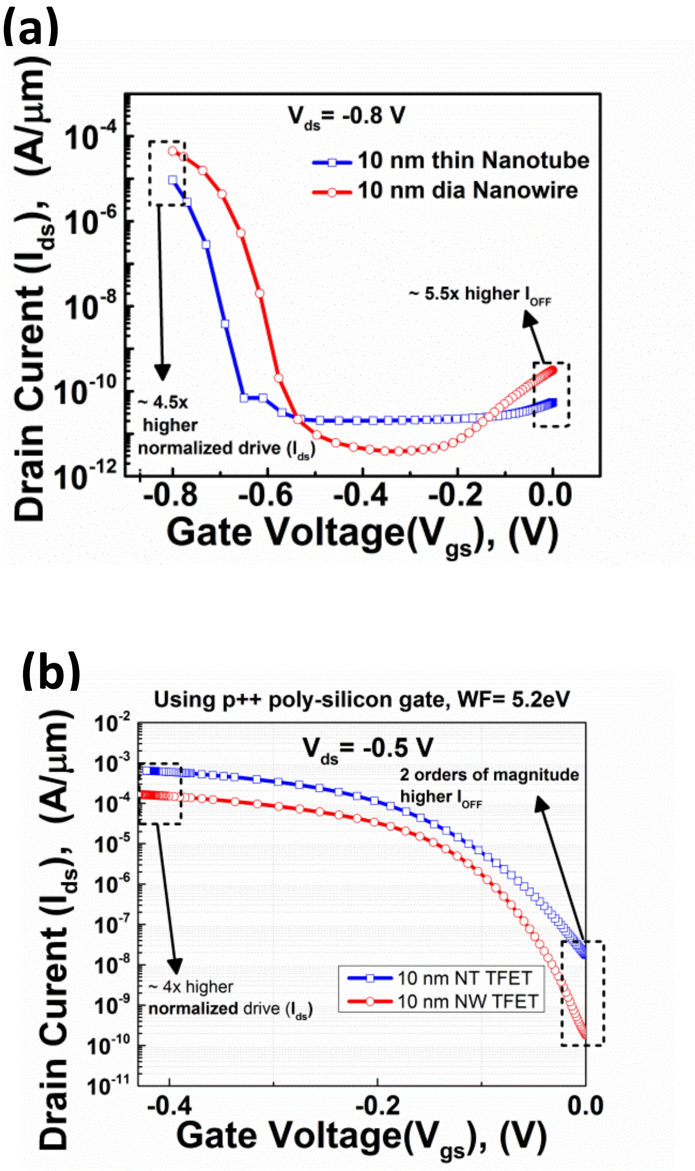

Figure S1| **(a)** Transfer characteristics at $V_{dd} = 0.8V$, and **(b)** $V_{dd} = 0.5V$ using heavily doped p^{++} polysilicon gate of 5.2eV work function.

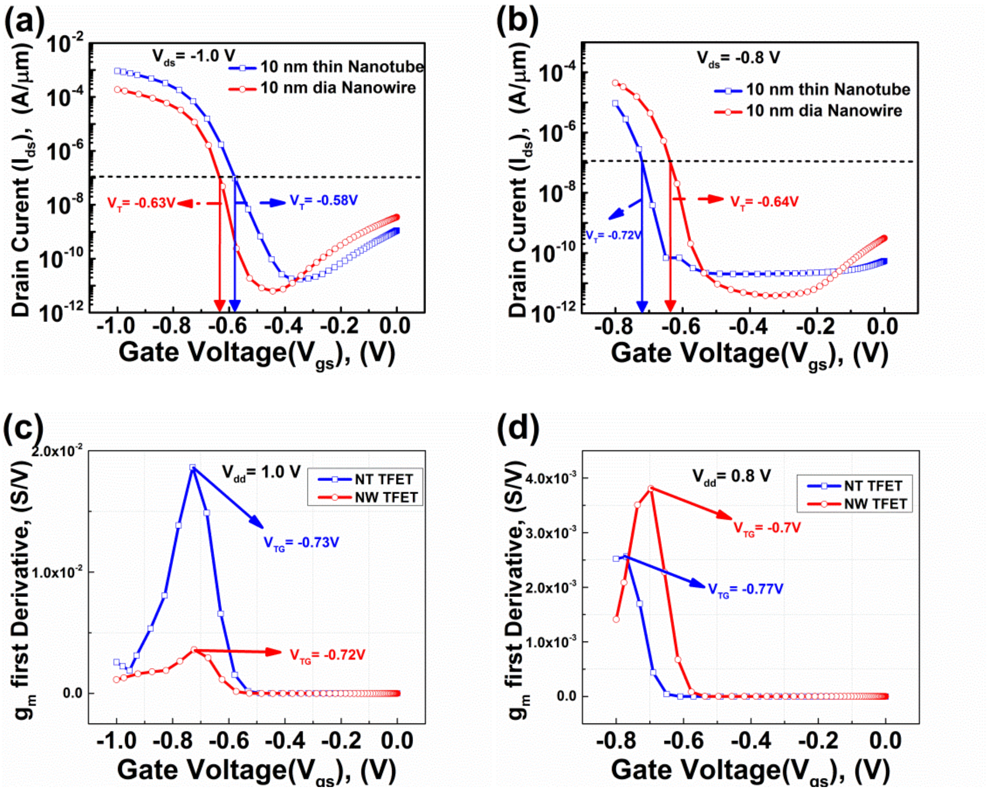

Figure S2| Transfer curve of 10 nm NT and NW TFETs showing VT using constant current method at I_{ds} = 10^{-7} A/μm at (a) V_{dd} = 1.0V and (b) V_{dd} = 0.8V. V_{TG} calculated using the first derivative of the normalized transconductance μm at (c) V_{dd} = 1.0V and (d) V_{dd} = 0.8V.

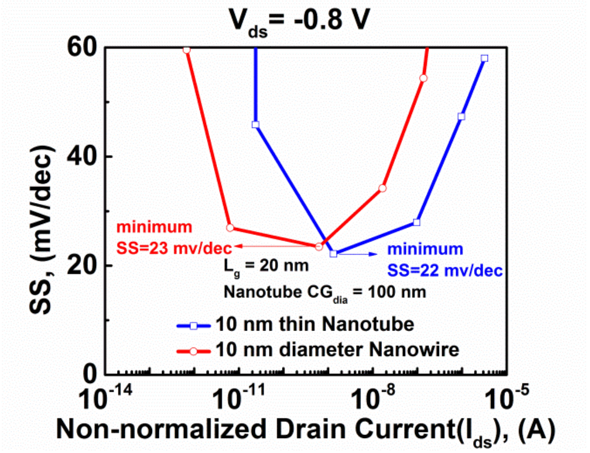

Figure S3| Subthreshold slope, SS, *vs.* non-normalized drain current for V_{dd}= 0.8V.

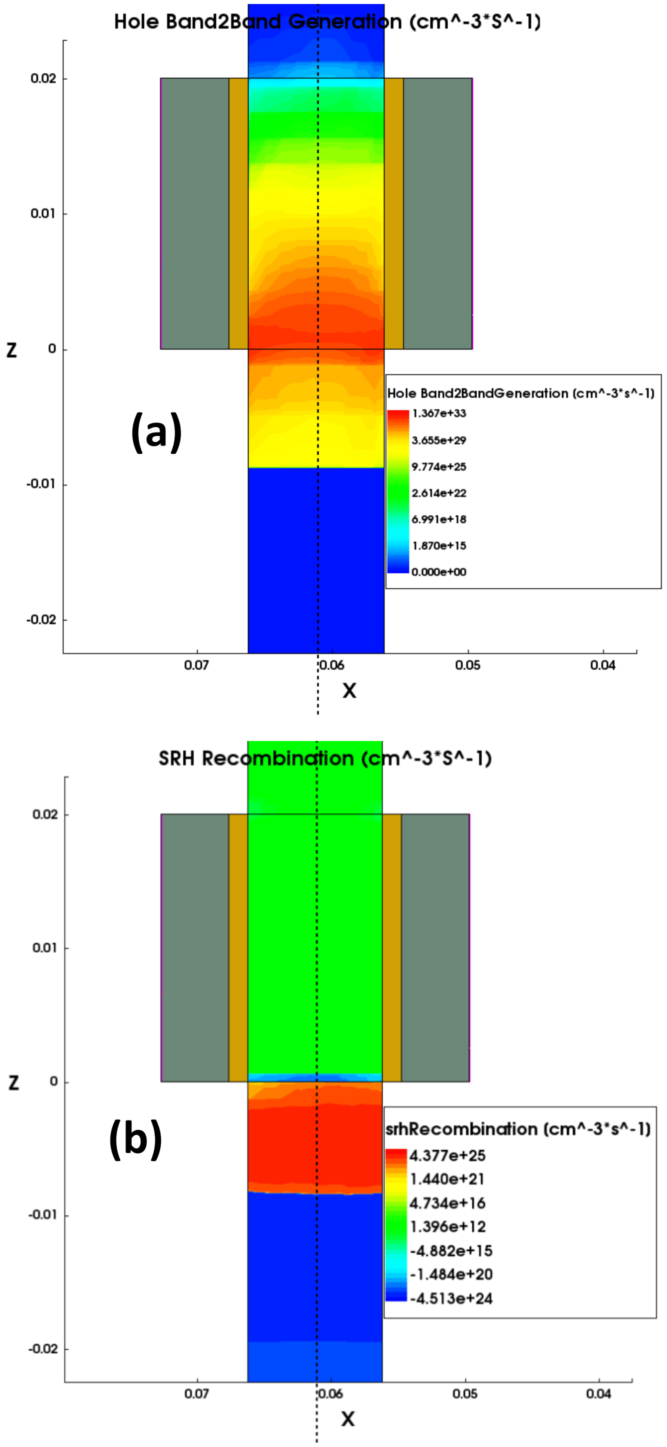

Figure S4| Color maps of 10 nm NT TFET **(a)** Hole Band-to-Band (B2B) Generation rate, and **(b)** SRH Recombination rate. X sections are indicated where the B2B generation and SRH recombination rates are measured in (Figure 7).

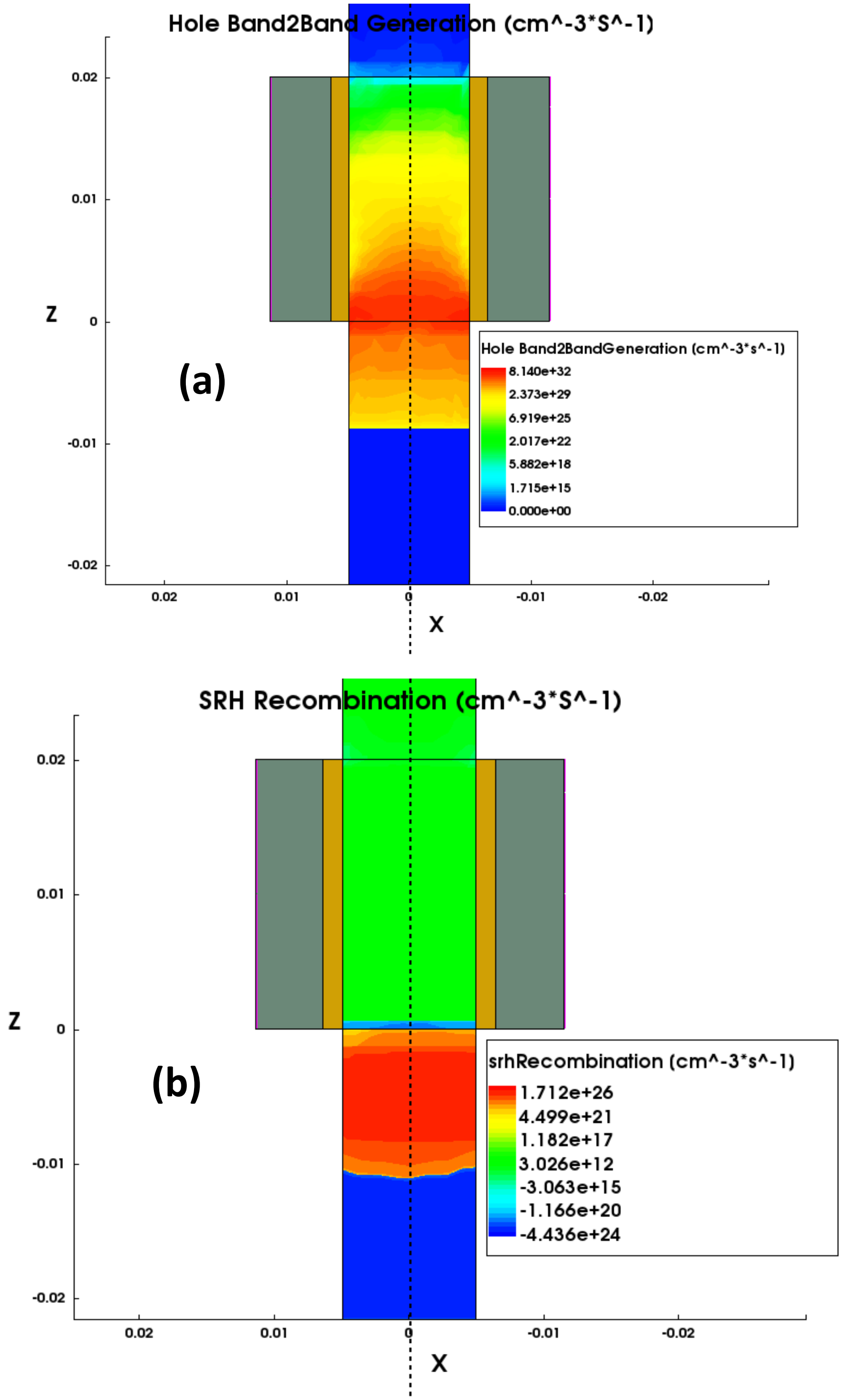

Figure S5| Color maps of 10 nm NW TFET**(a)** Hole Band-to-Band Generation rate, and **(b)** SRH Recombination rate.

X sections are indicated where the B2B generation and SRH recombination rates are measured in (Figure 7).

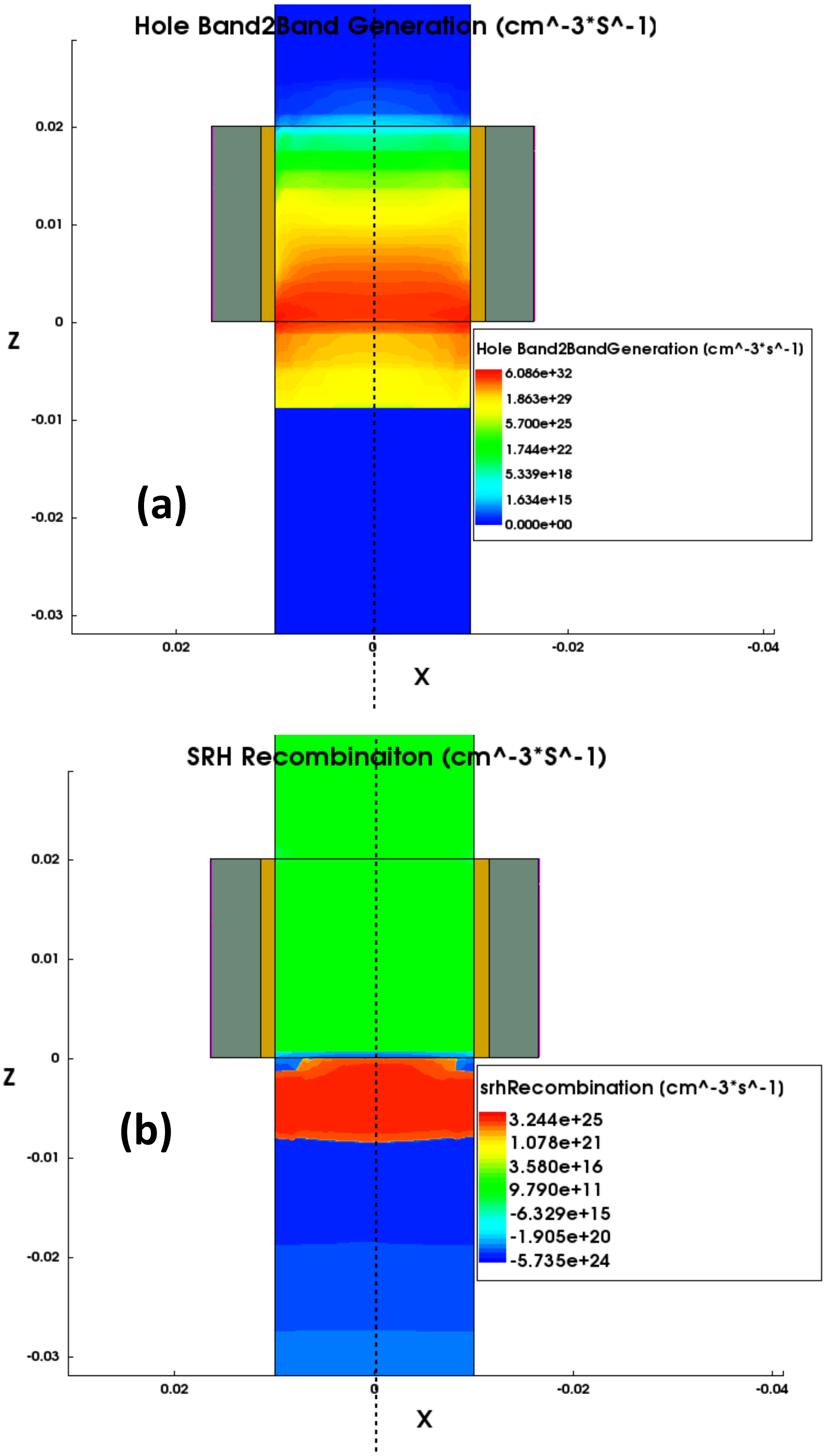

Figure S6| Color maps of 20 nm NW TFET**(a)** Hole Band-to-Band Generation rate, and **(b)** SRH Recombination rate. X sections are indicated where the B2B generation and SRH recombination rates are measured in (Figure 7).

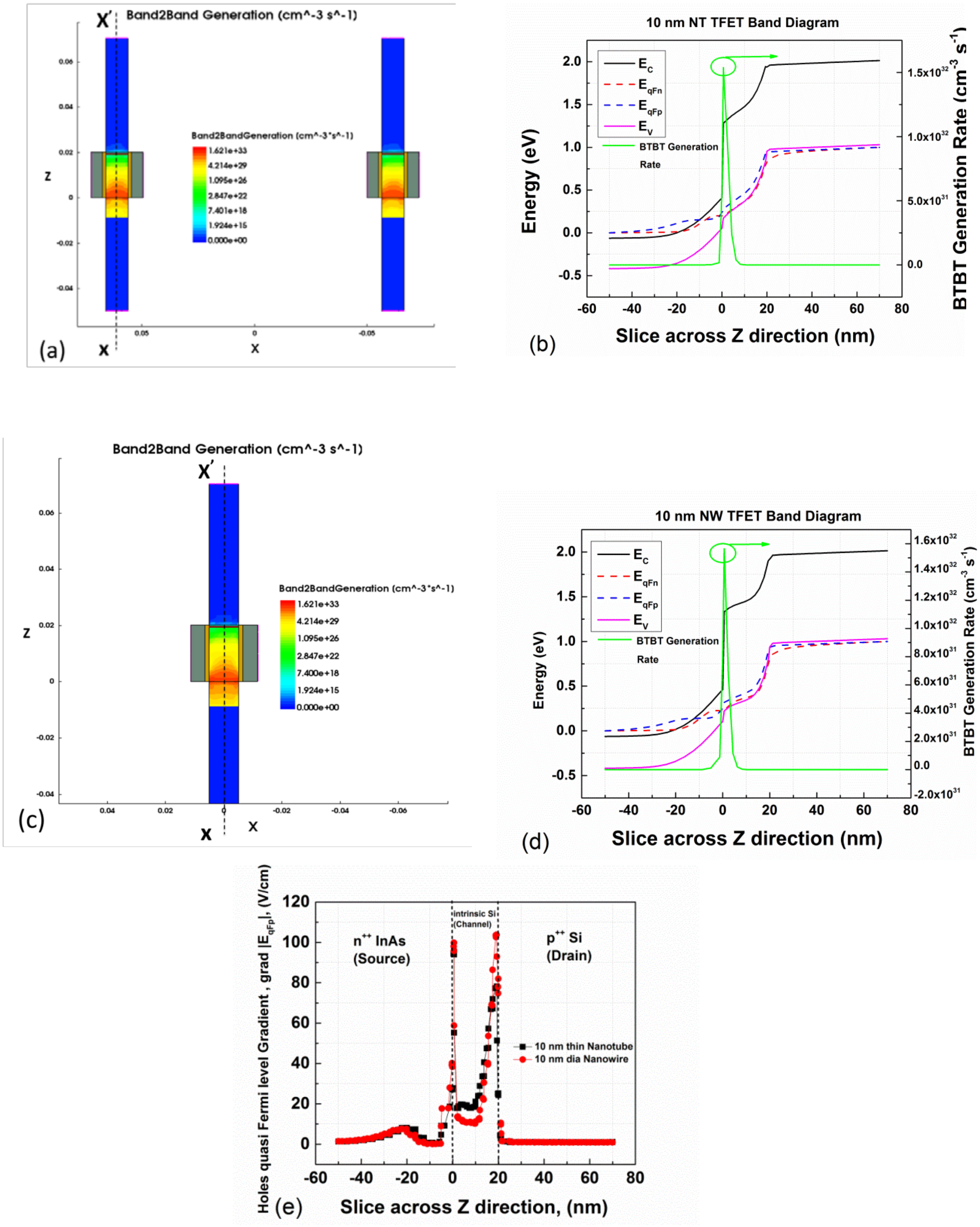

Figure S7| Color maps of 10 nm NT **(a)** and 10 nm NW **(c)** TFETs hole BTBT Generation rates, and **(b, d)** band diagrams and BTBT generation rate along the **x-x'** cross section in the middle of the channel for the NT and NW, respectively. **(e)** Absolute value of the gradient of hole quasi Fermi levels $|grad(E_{qFp})|$, for the 10 nm NT and 10 nm NW TFETs for the **x-x'** cross sections shown in **(b, d).**
